# Synthesis, crystal structure, DFT studies and biological activity of (Z)-3-(3-bromophenyl)-1-(1,5-dimethyl-1*H*-pyrazol-3-yl)-3-hydroxyprop-2-en-1-one

**DOI:** 10.1186/s13065-018-0492-4

**Published:** 2018-11-26

**Authors:** Said Tighadouini, Redouane Benabbes, Monique Tillard, Driss Eddike, Khadija Haboubi, Khalid Karrouchi, Smaail Radi

**Affiliations:** 1 Laboratoire de Chimie Appliquée et Environnement (LCAE), Faculté des Sciences, Université Mohamed I, 60000 Oujda, Morocco; 2 Département de Biologie, Faculté des Sciences, Université Mohamed I, 60000 Oujda, Morocco; 3 ICGM, CNRS, Université de Montpellier, ENSCM, Montpellier, France; 4 Laboratoire de Chimie du Solide Minéral et Analytique, Faculté des Sciences, Université Mohamed I, 60000 Oujda, Morocco; 5 ENSA-AL Hociema, Université Mohamed I, 60000 Oujda, Morocco; 6 Laboratoire de Chimie Thérapeutique, Faculté de médecine et de Pharmacie, Université Mohamed V, Rabat, Morocco

**Keywords:** β-Keto-enol-pyrazole, Single-crystal structure, NBO analysis, Reactivity indices, Fukui and Parr functions, Biological activity

## Abstract

**Background:**

Nowadays, is emerging a new generation of highly promising inhibitors bearing the β-ketoenol functionality. The present work relates to the first synthesis, the structure determination, the DFT studies and the use of a new biomolecule designed with a β-ketoenol group bounded to a pyrazolic moiety.

**Result:**

A novel β-ketoenol-pyrazole has been synthesized, well characterized and its structure was confirmed by single crystal X-ray diffraction. The electron densities and the HOMO–LUMO gap have been calculated using the DFT method with BLYP, PW91, PWC functionals and 6-31G* basis set. An evaluation of the molecule stability is provided by a NBO analysis and the calculated Fukui and Parr functions have been used to locate the reactive electrophile and nucleophile centers in the molecule. The synthesized compound, screened for its in vitro antifungal behavior against the *Fusarium oxysporum* f.sp. albedinis FAO fungal strains, shows a moderate activity with an inhibition percentage of 46%. The product was also tested against three bacterial strains (*Escherichia coli*, *Bacillus subtilis* and *Micrococcus luteus*), but no significant effect was observed against these organisms.

**Conclusions:**

Density functional calculations are used to evaluate the HOMO–LUMO energy gap, the molecular electrostatic potential and to provide a natural bond orbital analysis. The measured antimicrobial activities encourage us to continue searching for other structures, likely to be good antifungal candidates.
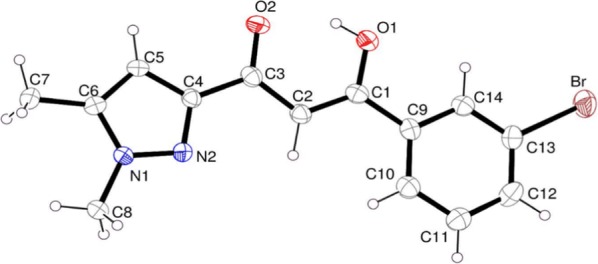

**Electronic supplementary material:**

The online version of this article (10.1186/s13065-018-0492-4) contains supplementary material, which is available to authorized users.

## Introduction

Pyrazoles represent a class of compounds endowed with a great interest in many domains. They have been widely described in the literature as chelating ligands [1–6] and several works have been gathered in reviews [7–10]. According to numerous literature reports, these derivatives are also well-known as important heterocyclic biologically active compounds, acting as antitumor [11], antiviral [12], anti-inflammatory [13] anti-anxiety [14] or antimicrobial [15] agents.

On the other hand, β-ketoenols form an important class of compounds, with an interest both in medical and pharmaceutical fields, regarded as drugs against HIV [16–18], cancer [19–22] and influenza [23] but also as antioxidant [24] and anti-inflammatory [25] substances. The β-ketoenol derivatives play also an important role in the development of coordination chemistry, as they are able to easily form stable complexes with most transition metals involving different modes of coordination and different functionalities [26, 27].

The pyrazoles associated with β-ketoenol groups lead to compounds with promising properties in both medicinal and coordination chemistry fields. In our recent works, some heterocycles containing the β-keto-enol functionality have been reported, that show significant biological activity [28] as well as interesting coordination properties [29–33].

The intention of this work was to develop of a novel pyrazole-based compound bearing a β-ketoenol functionality. Its crystal structure was solved from X-ray single crystal data and DFT studies were realized. The compound was also evaluated for its in vitro antifungal activity against *Fusarium oxysporum* f.sp. albedinis FAO fungal strains and against three bacterial strains (*Escherichia coli*, *Bacillus subtilis* and *Micrococcus luteus*).

## Results and discussion

### Chemistry

**Scheme 1:**
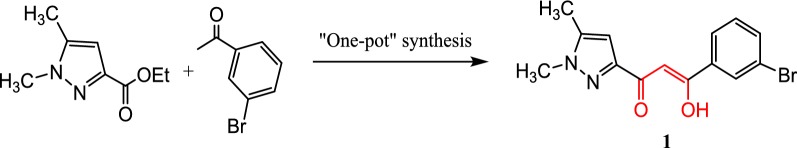
Synthesis of the target compound **1**

The target biomolecule based on β-ketoenol and pyrazole entities was prepared by a one-pot in situ condensation method which is similar to the procedures given in our previous works [28]. A solution of pyrazolic carboxylate was added to a suspension of sodium in toluene, then 1-(3-bromophenyl)ethanone was added at 0 °C (Scheme 1). After 2-days stirring at room temperature, the resulting precipitate has been treated and neutralized. The extracted organic layer was concentrated, dried and purified by silica gel column chromatography (see “Experimental section” part for details).

The β-keto-enol form was confirmed by the ^1^H-NMR analysis of the compound whose spectrum (Additional file 1: Figure S1) shows a strong signal assigned to the =C–H group of the keto-enol form at 6.54 ppm, it represents 85% of the compound. The diketone form is also present in a maximal proportion of 15% and was detected by the weak signal at 4.54 ppm which was attributed to the CH_2_ group of the diketone form. Traces of the keto form have also been detected in DEPTQ-135, which shows quaternary carbon atoms (C) and CH_2_ group as very small negative signals (Additional file 1: Figures S2 and S3). Good quality crystals of the major β-ketoenol structure were grown from methanolic solution by slow evaporation. The FT-IR spectrum confirms the formation of the ketoenol form with an enolic band at 1531 cm^−1^ (Additional file 1: Figure S4). Also in good agreement, the mass spectrum shows a molecular peak at 320.97. (Additional file 1: Figure S5).

### X-ray crystal structure description

Single crystals of (Z)-3-(3-bromophenyl)-1-(1,5-dimethyl-1H-pyrazol-3-yl)-3-hydroxyprop-2-en-1-one (**1**) were analyzed by X-ray diffraction in order to determine the compound structure.

The main crystal data are given with principal refinement parameters in Table 1 and the atom position and displacement parameters are listed in Table 2. The full CIF file deposited at the Cambridge Crystallographic Data Center (CCDC 1817604) is available at http://www.ccdc.cam.ac.uk/conts/retrieving.html.

**Table 1:** Crystal data and structure refinement for C_14_ H_13_ Br N_2_ O_2_

CCDC deposit number	1817604
Formula, M, Z	C_14_ H_13_ Br N_2_ O_2_, 321.17, 4
Space group	Triclinic, ${\text{P}}\bar{1}$
Lattice	a = 11.1458(7), b = 11.6337(3), c = 12.7221(9) Å,
	α = 112.075(2), β = 105.637(2), γ = 103.793(2)
θ range	1.88 to 29.09°
Reflections	18,366 collected/6297 unique [R(int) = 0.0414]
Crystal	colorless, 0.20 × 0.13 × 0.12 mm
Data/parameters	6297/357
R indices [I > 2σ(I)]	R1 = 0.0451, wR2 = 0.0968
R indices (all data)	R1 = 0.1012, wR2 = 0.1068
Δρ Fourier residuals	0.55/−0.33 e.Å^−3^

**Table 2:** Atomic coordinates (× 10^4^) and equivalent isotropic displacement parameters (Å^2^ × 10^3^) for C_14_ H_13_ Br N_2_ O_2_. U_eq_ is defined as 1/3 of the trace of the orthogonalized U_ij_ tensor

	Molecule 1				Molecule 2			
	x	y	z	Ueq	x	y	z	U_eq_
Br	56(1)	6869(1)	227(1)	66(1)	1104(1)	12,097(1)	− 3397(1)	77(1)
O1	− 4056(2)	6644(2)	− 3183(2)	56(1)	4307(2)	11,637(2)	182(2)	55(1)
O2	− 6166(2)	6129(2)	− 4913(2)	55(1)	5549(2)	11,122(2)	1789(2)	57(1)
N1	− 10,238(2)	3242(2)	− 5973(2)	38(1)	3715(3)	8399(2)	3102(2)	44(1)
N2	− 8973(2)	3673(3)	− 5150(2)	40(1)	3306(3)	8867(3)	2305(2)	44(1)
C1	− 4761(3)	5719(3)	− 3007(3)	40(1)	3195(3)	10,766(3)	28(3)	42(1)
C2	− 6118(3)	5006(3)	− 3717(3)	42(1)	3209(4)	10,106(3)	720(3)	44(1)
C3	− 6786(3)	5265(3)	− 4677(3)	42(1)	4436(3)	10,317(3)	1601(3)	42(1)
C4	− 8234(3)	4514(3)	− 5421(3)	37(1)	4448(3)	9595(3)	2341(3)	40(1)
C5	− 9038(3)	4600(3)	− 6420(3)	43(1)	5559(3)	9567(3)	3141(3)	46(1)
C6	− 10,317(3)	3787(3)	− 6759(3)	39(1)	5077(3)	8798(3)	3630(3)	39(1)
C7	− 11,613(3)	3477(4)	− 7736(3)	53(1)	5792(4)	8413(3)	4543(3)	55(1)
C8	− 11,320(3)	2284(3)	− 5949(3)	50(1)	2700(3)	7551(4)	3293(3)	62(1)
C9	− 3978(3)	5493(3)	− 2014(3)	41(1)	1964(3)	10,554(3)	− 957(3)	42(1)
C10	− 4596(3)	4618(3)	− 1630(3)	53(1)	700(3)	9632(3)	− 1272(3)	51(1)
C11	− 3823(4)	4409(4)	− 715(3)	61(1)	− 430(4)	9417(4)	− 2231(4)	61(1)
C12	− 2436(4)	5076(4)	− 175(3)	59(1)	− 292(4)	10,156(4)	− 2853(3)	65(1)
C13	− 1844(3)	5938(3)	− 548(3)	47(1)	950(4)	11,076(3)	− 2530(3)	51(1)
C14	− 2599(3)	6146(3)	− 1451(3)	43(1)	2073(3)	11,293(3)	− 1593(3)	46(1)

**Fig. 1:**
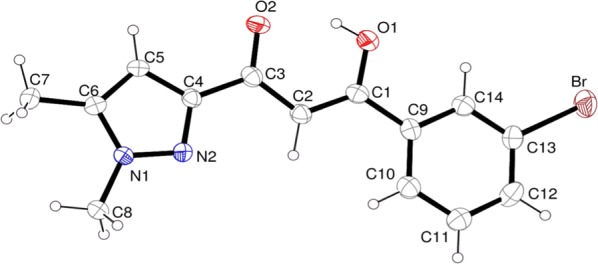
Ortep molecular representation of C_14_ H_13_ Br N_2_ O_2_ (30% probability ellipsoids)

The asymmetric unit contains two independent molecules. Each molecule is built with two rings, a bromophenyl ring bonded (at meta position) to a dimethyl pyrazole ring through a central core unit –CO–C–COH– (Fig. 1). According to the root mean square deviations of the fitted atoms in each group, ranging from 0.003 to 0.008, these three units are planar. The dihedral angles between the central core and the two rings, between 4.3 and 8.7°, indicate a slight deviation to flatness within each of the independent molecules. The two independent molecular units are almost coplanar, as shown by the angle of only 1.96(3)° measured between their mean planes (calculated with all the non-H atoms). The bonds lengths and angles measured in the two molecules are very close and in range of values found in the literature for similar compounds [34–38].

**Fig. 2:**
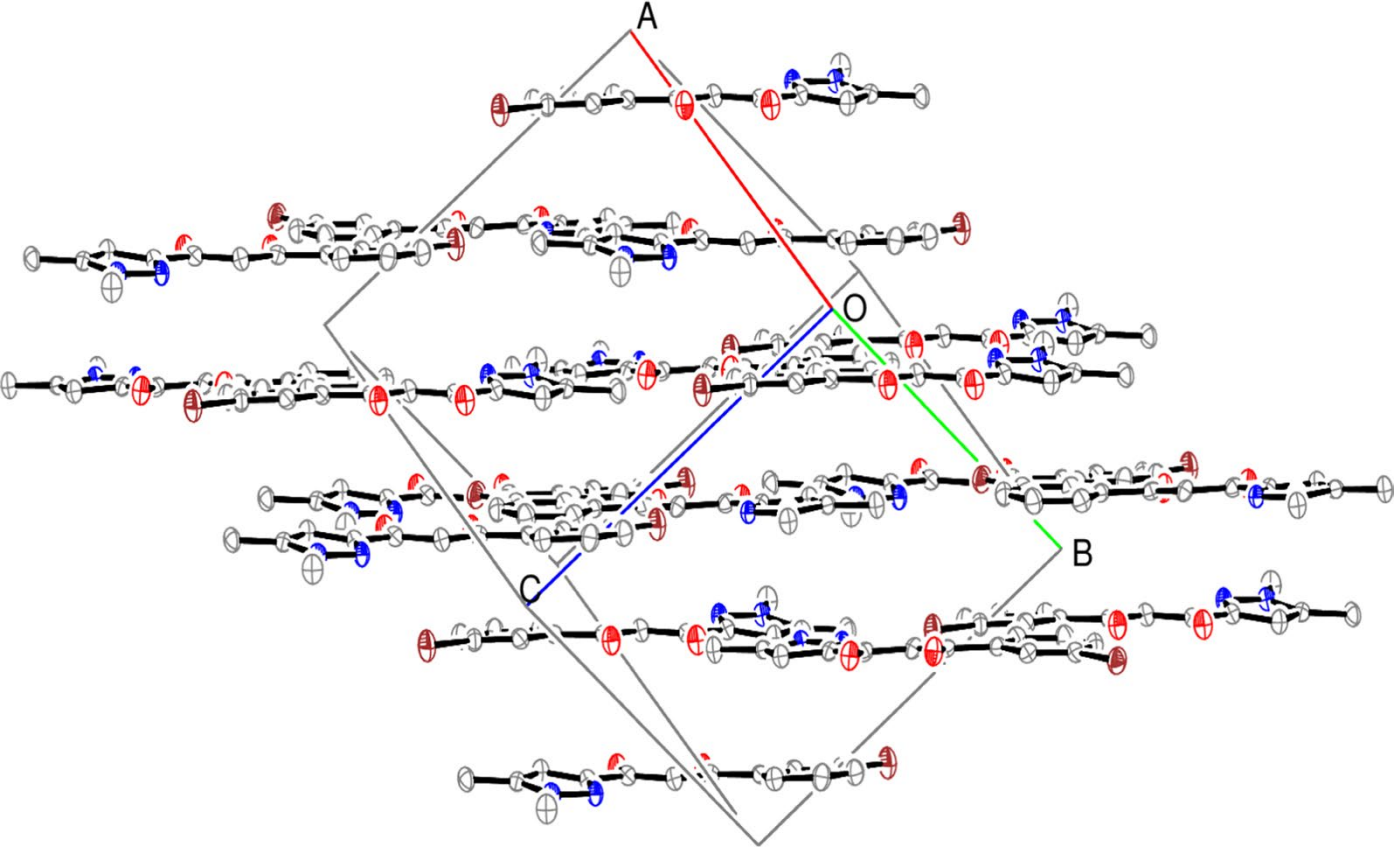
Molecular packing in the triclinic lattice enhancing the peculiar layered arrangement of molecules in planes parallel to ($1\bar{1}\bar{1}$)

The molecular arrangement in the solid is such as one unit cell contains four molecules, two-by-two symmetry-related. As a consequence of the co-planarity of the independent molecular units, the packing in the crystal results in a layered arrangement shown in Fig. 2. Within planes parallel to $\left( {1\bar{1}\bar{1}} \right)$ and separated by ~ 3.5 Å, each molecule is surrounded by six homologous units, this molecular organization is studied later in this work for evaluation of in-plane interactions. On the other hand, no π-stacking interaction must be expected because of relative position of the successive planes without ring overlapping.

### DFT calculations

To investigate the molecular geometry and the electron distribution in the solid, density functional theory (DFT) calculations were carried out using the program Dmol^3^ at three DFT levels: with PW91 or BLYP functional within the GGA (generalized gradient approximation) and with PWC functional within the LDA (local density approximation) [39–41]. Double numerical plus polarization DNP basis sets were taken in all calculations.

**Fig. 3:**
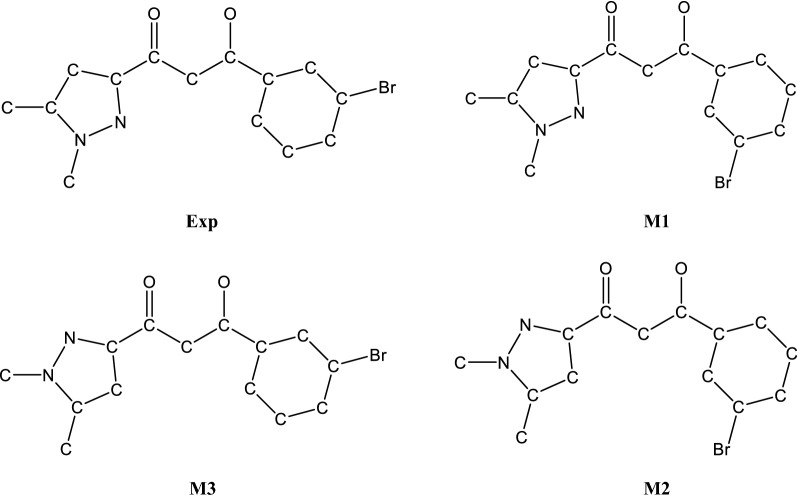
Experimental molecule and hypothetical models built by changing the rings orientation

Full geometry optimization (LDA-PWC) by minimization of the total energy was first carried out, both starting from experimental geometry and from three planar geometries built by rotation of the rings around the C1–C9 and C3–C4 bonds (Fig. 3).

The total energies calculated indicate a lower stability, by 0.16 and 0.17 eV, for the M2 and M3 models compared to experimental geometry. This potentially results from the occurrence of some N···O repulsive interactions in these configurations. Instead, the M1 model is almost as stable (only differing by 0.001 eV) as the experimental molecule in which the central core and the rings are rigorously coplanar (dihedral angles lower than 2°).

**Fig. 4:**
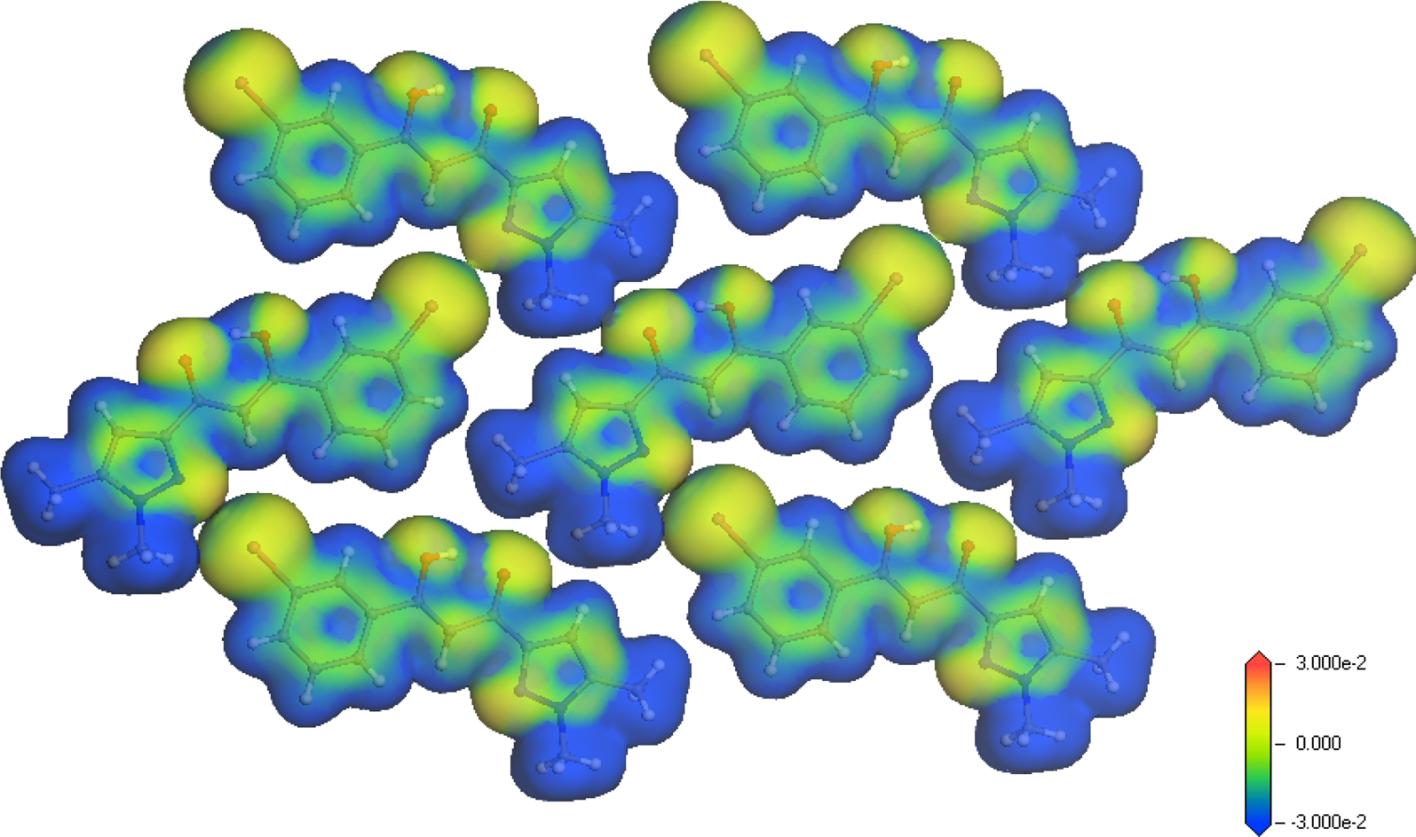
Representation of the 3D isosurface electron density (volumic contour) mapped with the deformation density. Positive value (yellow domains) indicate electron localization

Trying to evaluate intermolecular interactions within the solid, geometry was optimized for a large molecular fragment consisting of a molecule and its six surrounding neighbors. The three dimensional contour of the total density, drawn at the 0.04e^−^/Å^3^ isolevel and mapped with the electron deformation density, has been represented in Fig. 4. The deformation density, computed as the total electron density with the density of isolated atoms subtracted, points outs the electron localization as positive regions and the electron losses as negative regions. Looking at its representation, one can conclude that no strong intermolecular interaction exists in this compound. Nevertheless, the positive domains (yellow zones in Fig. 4) indicate the occurrence of an interaction between the bromine atom of a molecule and the nitrogen N2 atom of a neighboring molecule.

As expected, the periodic calculations in the solid confirm the absence of any bonding density between the molecular planes separated by 3.5 Å. Selected bonds and angles given in Table 3 illustrate the structural, and rather weak, packing constraints onto the molecular geometry. It is interesting to remark that the dihedral angle between mean planes of the two independent molecules in the unit cell is about 4° in the optimized structures, a value which is nearly twice the angle in the experimental crystal. On the other hand, the experimental torsion angles C4–C3–C2–C1 and C3–C2–C1–C9, which reflect the molecule flatness, range from 176.8 to 179.6° and do not differ much from the values in optimized molecular (177.8–179.9°) and 3D periodic (175.6–179.8°) models.

**Table 3:** Selected bond lengths [Å] and angles [°] for C_14_ H_13_ Br N_2_ O_2_

	Mole 1/mole 2	PW91	BLYP	PWC	Solid PWC	Solid BLYP	Solid PW91
Br–C13	***1.900(3)/1.910(3)***	*1.928*	*1.955*	*1.899*	*1.913*–*1.916*	*1.950*–*1.957*	*1.928*–*1.935*
O2–C3	***1.260(4)/1.262(3)***	*1.275*	*1.276*	*1.280*	*1.282*–*1.283*	*1.281*–*1.280*	*1.280*–*1.277*
N1–N2	***1.345(3)/1.348(3)***	*1.344*	*1.358*	*1.326*	*1.328*–*1.330*	*1.353*–*1.357*	*1.341*–*1.345*
N1–C6	***1.365(4)/1.361(4)***	*1.378*	*1.386*	*1.365*	*1.364*–*1.365*	*1.379*–*1.381*	*1.373*–*1.375*
N1–C8	***1.450(4)/1.452(4)***	*1.451*	*1.464*	*1.431*	*1.434*–*1.435*	*1.461*–*1.463*	*1.449*–*1.451*
N2–C4	***1.333(4)/1.329(4)***	*1.353*	*1.336*	*1.338*	*1.342*–*1.341*	*1.356*–*1.354*	*1.351*–*1.350*
O1–C1	***1.304(4)/1.312(4)***	*1.328*	*1.343*	*1.297*	*1.303*–*1.301*	*1.342*–*1.342*	*1.328*–*1.328*
N2–N1–C6	***112.8(2)/113.5(2)***	*112.3*	*112.7*	*111.3*	*113.2*–*113.1*	*112.9*–*112.7*	*113.1*–*113.2*
C4–N2–N1	***104.3(2)/103.8(2)***	*104.7*	*104.7*	*104.8*	*104.9*–*105.0*	*104.6*–*104.8*	*104.8*–*104.7*
mol1–mol2 angle	***1.96***				*4.4*	*3.5*	*4.2*

Experimental (bolditalic) values in the crystal are compared with values calculated (italic) in molecular or 3D models

Within the overall context of the FMO theory, the energetic level and the form of the frontier orbitals are relevant parameters for analysis of the molecular reactivity [42]. Regardless of the DFT level of theory, an HOMO–LUMO energy separation of about 2.3 eV has been calculated with DMol^3^ for the molecule (2.7 eV in the crystal), this value should be compared with the gap of 3.46 eV measured experimentally from UV experiments (absorption peak at 358 nm) (Additional file 1: Figure S6). Both orbitals display a π-type character mainly localized on the central part of the molecule, which is bonding at the HOMO and antibonding at the LUMO levels.

### Fukui and Parr functional analysis

Calculated electron deformation density is strongly related to molecular electrostatic potential and for this reason may be equally used to discuss the reactivity [43]. The latter is also seen as a useful tool in evaluation of the regiochemistry, especially in reactions that are dominated by electrostatic effects. The electrophilic f(−) and nucleophilic f(+) Fukui functions, whose extreme values reflect the ability for an electrophilic or a nucleophilic attack, are defined as electron density derivatives with respect to the number of electrons at a constant potential. They can give a measure of the local reactivity and they have been considered suitable to rationalize the regioselectivity [44–47].

Though, it has been stated that regioselectivity in polar reactions should be predictable alternately using the local electrophilicity [48]. Then electrophilic and nucleophilic Parr functions P_k_^+^ and P_k_^−^ are powerful tools to study the molecular reactivity and they are well adapted to localize the electrophile and nucleophile centers in an organic molecule [49, 50]. They have been computed from the atomic spin density (difference between α and β electron densities) for the radical anion and the radical cation having the geometry optimized for the neutral molecule.

**Fig. 5:**
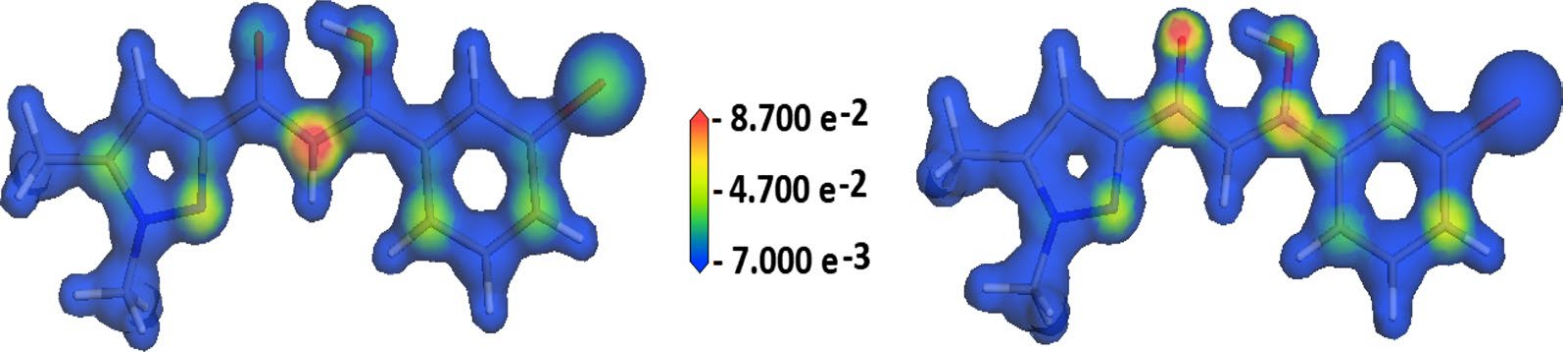
Atomic spin density spatial distribution mapped onto the electrostatic potential showing the electrophilic (left image) and nucleophilic (right image) centers as red zones

The spatial distribution of the atomic spin density mapped on the electrostatic potential provides a graphical view of the localization of electrophilic and nucleophilic centers (Fig. 5). In the present case, the Parr functions quite well validate the results predicted by Fukui functions with an electrophile center at C2 atom (and at a less extent at N2 and C10 atoms) while the nucleophile center is situated at the central core of the molecule mainly at O2 atom, most likely to undergo a nucleophilic attack.

The global electrophilicity index ω, expressed as μ^2^/2η [51], takes here the value of 2.28. It has been obtained from the electronic chemical potential μ (μ = 1/2 × (E_HOMO_ + E_LUMO_) = –4.37 eV) and the chemical hardness η (η = E_LUMO _− E_HOMO_ = 4.17 eV), both calculated with G03W code from the one-electron energies of the frontier orbitals. The maximal charge transfer (− μ/η) is found close to unity and the nucleophilicity index N close to 3. The latter is calculated comparatively to TCE taken as a reference from N = E_HOMO_ − E_HOMO_(TCE) where E_HOMO_(TCE) = 9.4083 eV is the energy calculated in the same conditions for tetracyanoethylene. Based on these calculated theoretical reactivity indices, the molecule is characterized with a moderate nucleophile and rather good electrophile character.

### Natural bond orbital (NBO) analysis

The NBO analysis is a helpful way to study the interactions among bonds and to examine the charge transfer resulting of conjugative interactions in a molecular system [52, 53]. The hyperconjugative interaction energy is deduced from a second-order perturbation approach [53]. Considering a donor (*i*) and an acceptor (*j*), the stabilization energy *E*(2) associated with delocalization is estimated as *q*_*i*_ × *F*(*i*, *j*)^2^/(*ε*_*i*_ − *ε*_*j*_), where *q*_*i*_ is the donor orbital occupancy, *ε*_*i*_ and *ε*_*j*_ are diagonal elements (orbital energies) and *F*(*i*, *j*) is the off-diagonal NBO Fock matrix element. The larger the stabilization energy, the stronger the donor-to-acceptor interaction, i.e. more important is the electron-donor trend towards acceptor and greater is the extent of conjugation on the whole system. Table 4 summarizes the highest interactions between bonding and antibonding (Lewis/non-Lewis) natural orbitals as for example between the π C1–C2 donor and the π* O2–C3 acceptor with a stabilizing energy of 32.54 kcal.mol^−1^. The lone pair (LP) orbitals are also seen to have important stabilizing contributions as illustrated with interaction of the lone pair at O1 atom with the π* C1–C2 acceptor (44.40 kcal.mol^−1^).

**Table 4:** Second-order perturbation analysis interactions in C_14_ H_13_ Br N_2_ O_2_

Donor (*i*)			Acceptor (*j*)			*E*(2)	*ε*_*i*_− *ε*_*j*_	*F*(*i*, *j*)
NBO type occupation			NBO type occupation			kcal.mol^−1^	a.u.	a.u.
O1	LP	1.79161	C1–C2	π*	0.25875	44.40	0.36	0.114
N1	LP	1.52345	C5–C6	π*	0.32998	38.56	0.31	0.101
C1–C2	π	1.76788	O2–C3	π*	0.32146	32.54	0.28	0.087
N1	LP	1.52345	N2–C4	π*	0.46366	31.65	0.28	0.084
C5–C6	π	1.76975	N2–C4	π*	0.46366	29.85	0.27	0.085
C2–H2	σ	1.96995	C13–C14	π*	0.35711	22.20	4.36	0.304
C11–C12	π	1.65057	C9–C10	π*	0.37373	21.06	0.29	0.070
C9–C10	π	1.62726	C11–C12	π*	0.32589	20.12	0.28	0.068
C13–C14	σ	1.67945	C11–C12	π*	0.32589	20.03	0.30	0.070
C13–C14	σ	1.67945	C9–C10	π*	0.37373	17.75	0.30	0.067
O2	LP	1.90100	C3–C4	σ*	0.05429	16.30	0.75	0.100
N2–C4	π	1.83133	O2–C3	π*	0.32146	15.55	0.31	0.064
C9–C10	π	1.62726	C1–C2	π*	0.25875	14.87	0.29	0.060
N2–C4	π	1.83133	C5–C6	π*	0.32998	13.19	0.34	0.062
O2	LP	1.90100	O1–HO1	σ*	0.03204	12.68	1.12	0.108
O2	LP	1.90100	C2–C3	σ*	0.04207	12.25	0.80	0.090
C1–C2	π	1.76788	C9–C10	π*	0.37373	9.46	0.29	0.049

E(2) is the hyper conjugative interaction energy, *εi *− *εj* the energy difference between *i* and *j* NBO orbitals and F(*i, j*) the Fock matrix element between *i* and *j* NBO orbital

*LP* lone pair

### Biological activity

**Fig. 6:**
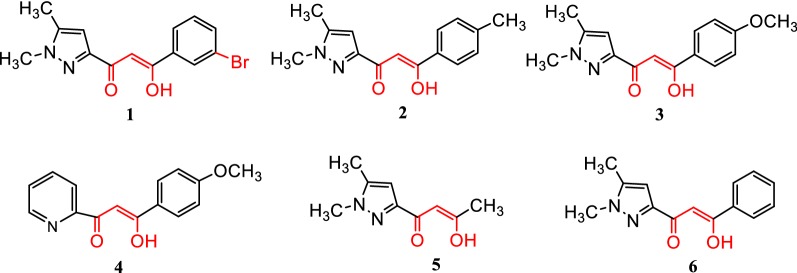
Molecular structures of compounds **1**–**6**

The in vitro antibacterial and antifungal activities of compound **1** were tested by the agar diffusion technique [54–56] using fungal strains (*Fusarium oxysporum* f.sp. albedinis *FAO*) and bacterial strains (*Escherichia coli*, *Bacillus subtilis*, and *Micrococcus luteus*). Tests were also performed for comparison on several compounds already prepared in our former works (Fig. 6).

Results of the tests carried out towards bacterial strains for the new compound **1** and also for the other products **2**–**6** revealed no significant effect against these organisms.

On the contrary, the structure **1** led to a moderate inhibiting antifungal activity of 46% occurring at 500 μL of sample. This antifungal effect remains rather modest with regard to the benomyl fungicide (94%) as reported in Table 5.

**Table 5:** Values of antifungal activity of the pyrazole-keto enol compounds against *Fusarium oxysporum* f.sp.

Compounds	Volume withdrawn (µL)	Diameter of the strain in the presence of the drug (cm)	Inhibition (%)
**1**	50	5	0
	200	3.8	24
	500	2.7	46
**2**	50	5	0
	200	3.5	30
	500	2.3	54
**3**	50	5	0
	200	3.6	28
	500	2.5	50
**4**	50	5	0
	200	3.8	24
	500	3.2	36
**5**	50	1.2	76
	200	0.9	82
	500	0.5	*90*
**6**	50	2.0	60
	200	1.3	74
	500	0.2	*96*
Benomyl	50	2.3	54
	200	1.1	78
	500	0.3	*94*

Generally, the results obtained for this new structure are in agreement with activities of the similar compounds **2**–**4** [28]. The inhibiting percentage of 46% found for **1** is in the range of values obtained for compounds **2**–**4**, between 36 and 54%. Instead, the antifungal performances found for structures **5** and **6**, also belonging to the same family, reach values very close to the benomyl fungicide taken as reference. Such differences in the biological activity are obviously dependent on the radical group attached to the pyrazol ketoenol fragment. The best inhibiting activities are obtained with a methyl or a phenyl group while the presence of substituted phenyl groups (*m*-bromophenyl, *p*-methyl phenyl or *p*-metoxy phenyl) provide lower antifungal properties. These findings push us to dig deeper to find much more interesting molecules. Of course, various structural modifications to bring to these compounds as antifungal candidates are currently in progress.

## Experimental section

### General information

All solvents and other chemicals (purity > 99.5%, Aldrich, Saint-Louis, MO, USA) of analytical grade were used without further purification. An Xcalibur four circle CCD diffractometer (Oxford Diffraction, Abingdon, Oxfordshire, England) was used to collect the X-ray intensities diffracted by a parallelepiped colorless selected crystal (CNRS, ICGM, France). Elemental analyses were performed by the Microanalysis Centre Service (CNRST, Rabat, Morocco). Melting points were measured using a Büchi 510 m.p. apparatus (LCAE, Oujda, Morocco). ^1^H and ^13^C-NMR spectra were recorded using an AC 300 spectrometer (CNRST) (Bruker, LLN, Belgium) (300 MHz for ^1^H and 75.47 MHz for ^13^C spectra). A JMS DX-300 mass spectrometer (JEOL, Rabat, Morocco) was used for the determination of molecular weights. Infrared (IR) spectra were recorded on a Shimadzu infrared spectrophotometer (LCAE, Oujda, Morocco) using the KBr disc technique. Geometry optimizations and DFT calculations were carried out using Dmol^3^ and Gaussian G03W programs (CNRS, ICGM, France).

### Procedure for the synthesis of pyrazole-ketoenol

To a solution of toluene (20 mL) containing metallic sodium (15.21 mmol) was added the pyrazolic carboxylate (12.01 mmol) solubilized in toluene (20 mL); then 3-bromophenyl methyl ketone (12.01 mmol) in toluene (10 mL) was added at 0 °C. The resulting mixture was stirred at room temperature for 2 days. The resulting precipitate was filtered, washed, dissolved in water, and neutralized with acetic acid to pH 5. The CH_2_Cl_2_ extracted fraction was dried over anhydrous sodium sulfate and concentrated to dry. The final product, as a white solid, was obtained after purification through silica gel column chromatography using CH_2_Cl_2_/MeOH in 28% yield. The β-ketoenol form was recrystallized from methanol (95%) to obtain the (Z)-3-(3-bromophenyl)-1-(1,5-dimethyl-1H-pyrazol-3-yl)-3-hydroxyprop-2-en-1-one compound which was confirmed by FT-IR, ^1^H-NMR, ^13^C NMR, and mass spectroscopy. yield: 28%; m.p. 124 °C; Rf = 0.52 (CH_2_Cl_2_/MeOH 9/1)/silica. IR (KBr, cm^−1^): ν (OH) = 3431; ν (C=O) = 1676; ν (enolic C=C) = 1531; ^1^H NMR [CDCl_3_, δ(ppm)]: 2.24 (s, 3H, Pz-CH_3_); 3.78 (s, 3H, CH_3_–N); 4.54 (s, 0.1H, keto, CH_2_); 6.54 (s, 0.9H, enol, C–H); 7.39 (m, 3H, Ar-2H, Pz–H); 7.91(m, 2H, Ar–H). ^13^C NMR [CDCl_3_, δ(ppm)]: 11.29 (1C, Pz-CH_3_); 36.88 (1C, CH_3_–N); 49.39 (1C, keto CH_2_); 93.24 (1C, enol C–H); 106.14 (1C, =CH, Pz); 127.01 (1C, Ar–C3); 128.53 (1C, Ar–C6); 132.04 (1C, Ar–C5); 133.31(1C, Ar–C2); 134.97 (1C, Ar–C1); 140.37 (1C, Ar–C4); 147.72 (2C, PzN=C, PzN–C=); 181.99 (1C, C–OH); 183.19 (1C, C=O). Anal. Calcd. for C_14_H_13_BrN_2_O_2_: C 52.36, H 4.08, N 8.72. Found: C 52.25, H 4.12, N 8.65. *m/z*: 320.97.

### X-ray data collection and treatment

Fairly regularly shaped crystals were selected using a stereomicroscope equipped with a polarizing filter. Diffracted intensities were collected at room temperature within the complete diffraction sphere on the four-circle diffractometer (Mo-Kα radiation, λ = 0.71073 Å) and data reduction was carried out using CrysAlis software [57]. The lattice dimensions and corresponding standard deviations were determined by least-squares method from the entire data set of reflections. Full-matrix least-squares refinements on F^2^ used the complete data set of 18,366 collected reflections (including symmetry equivalent and redundant) of which 6297 are unique and 3027 observed according to the criterion I > 2σ(I). The diffracted intensities were corrected for Lorentz and polarization effects. The structure solution and subsequent refinements were performed using SHELX-2013 program packages [58]. Atoms positions and anisotropic displacement parameters were refined for all non-hydrogen atoms. The hydrogen atoms at the keto enol OH group were detected in the final Fourier difference and were treated as riding, following the HFIX/AFIX instructions, in the final refinement (even if they could have been freely refined). The hydrogen atoms have been considered with an isotropic displacement parameter equal to 1.2 times (1.5 for terminal –CH_3_) the Ueq of the parent atom. Molecular pictures are drawn with ORTEP-3 for windows [59].

Full CIF file can be obtained free of charge via http://www.ccdc.cam.ac.uk/conts/retrieving.html (or from the CCDC, 12 Union Road, Cambridge CB2 1EZ, UK; Fax: +44 1223 336033; E-mail: deposit@ccdc.cam.ac.uk).

### Computation details

Calculations were performed within the framework of the density functional theory DFT using DMol^3^ module [60, 61] provided in Materials Studio software. A DNP basis set (Double Numerical with extra Polarization function on all atoms) was used in these calculations, its size is equivalent to the Gaussian 6-31G*. All-electrons geometry optimizations were performed at fine quality level, for both the molecule and the periodic crystal packing, with LDA-PWC, GGA-PW91 and GGA-BLYP functionals. The Fukui and Parr functions have been computed to give a description of the global reactivity. Gaussian 03 W optimizations using the Berny analytical gradient method with B3LYP functional and 6.31G+(d,p) basis set have been done prior to calculate the reactivity indices and to perform the NBO analysis [53].

### Anti-fungal tests

In vitro antibacterial and antifungal activities were tested by the agar diffusion technique (ADT) [54, 55]. ADT has been investigated using susceptibility test of NCCLS (National Committee for Clinical Laboratory Standards) recommended by the WHO and the French standard NF-U-47-107 AFNOR 2004. The agar media were inoculated with test organisms and a solution of the tested compound in DMSO/EtOH (50/50) was added to different concentrations in the culture media. The growth was followed by counting the bacteria or the yeast colonies and by measuring the mycelium diameter. The inhibition percentage of a molecule is equal to the ratio of the colonies number (or the mycelium diameter of the culture) in the presence of a dose of the tested compound over the colonies number (or the mycelium diameter) of the reference culture, multiplied by 100. The minimum inhibition concentration (MIC) is the least dose of the compound which causes an inhibition of the micro-organism growth. Calculation of the concentration IC_50_ was done using the same bacterial inocula mentioned above with decreasing concentration of the tested products. The D^o^ value was measured for each culture at 625 nm. The inhibition percentage (%) is expressed as (D° − Dx)/D° × 100 where D° is the diameter of the mycelial growth of the culture witness and Dx the diameter of the mycelial growth in the presence of the product to be tested.

## Conclusion

A novel pyrazole-β-ketoenol compound has been synthesized, it has been characterized by NMR and IR techniques and its XRD single crystal structure was determined. Density functional calculations are used to evaluate the HOMO–LUMO energy gap, the molecular electrostatic potential (MEP) and to provide a natural bond orbital (NBO) analysis. From reactivity indices, the present molecule displays a moderate electrophile character. Computed Parr functions quite well agree with Fukui functions, indicating the position of the nucleophile and electrophile centers in the molecule. The title compound has been tested against *Fusarium oxysporum* f.sp. albedinis FAO fungal strains and three bacterial strains (*Escherichia coli*, *Bacillus subtilis*, and *Micrococcus luteus*). The measured activities encourage us to continue searching for other structures, likely to be good antifungal candidates.

## Additional file

**Additional file 1: Figure S1.** ^1^H NMR spectrum of **1**, **Figure S2.** ^13^C NMR spectrum of **1**. **Figure S3.** ^13^C NMR-DEPTQ-135 spectrum of **1**. **Figure S4.** Mass spectrum of **1**. **Figure S5.** FT-IR spectrum of **1**. **Figure S6.** UV-Vis spectrum of **1**.

## Abbreviations

DFT: density functional theory
NBO: natural bond orbital
MEP: molecular electrostatic potential
HOMO: highest occupied molecular orbital
LUMO: lowest unoccupied molecular orbital
HIV: human immunodeficiency virus
LDA: local density approximation
DNP: double numerical plus polarization
