# Supplementary material for: Synthesis, crystal structure, DFT studies and biological activity of (Z)-3-(3-bromophenyl)-1-(1,5-dimethyl-1H-pyrazol-3-yl)-3-hydroxyprop-2-en-1-one
Source: Chem Cent J. 2018 Nov 26;12:122. doi: 10.1186/s13065-018-0492-4 (PMC6768133; doi:10.1186/s13065-018-0492-4)
Supplement: Supplementary file 1 — Additional file 1: Figure S1. 1H NMR spectrum of 1, Figure S2. 13C NMR spectrum of 1. Figure S3. 13C NMR-DEPTQ-135 spectrum of 1. Figure S4. Mass spectrum of 1. Figure S5. FT-IR spectrum of 1. Figure S6. UV-Vis spectrum of 1. [file 13065_2018_492_MOESM1_ESM.doc]

**Additional material**

# Synthesis, Crystal Structure, DFT Studies and Biological Activity of (Z)-3-(3-bromophenyl)-1-(1,5-dimethyl-1*H*-pyrazol-3-yl)-3-hydroxyprop-2-en-1-one

**Said Tighadouini, Redouane Benabbes,** **Monique Tillard*, Driss Eddike, Khadija Haboubi, Khalid Karrouchi*,** **Smaail Radi**

**
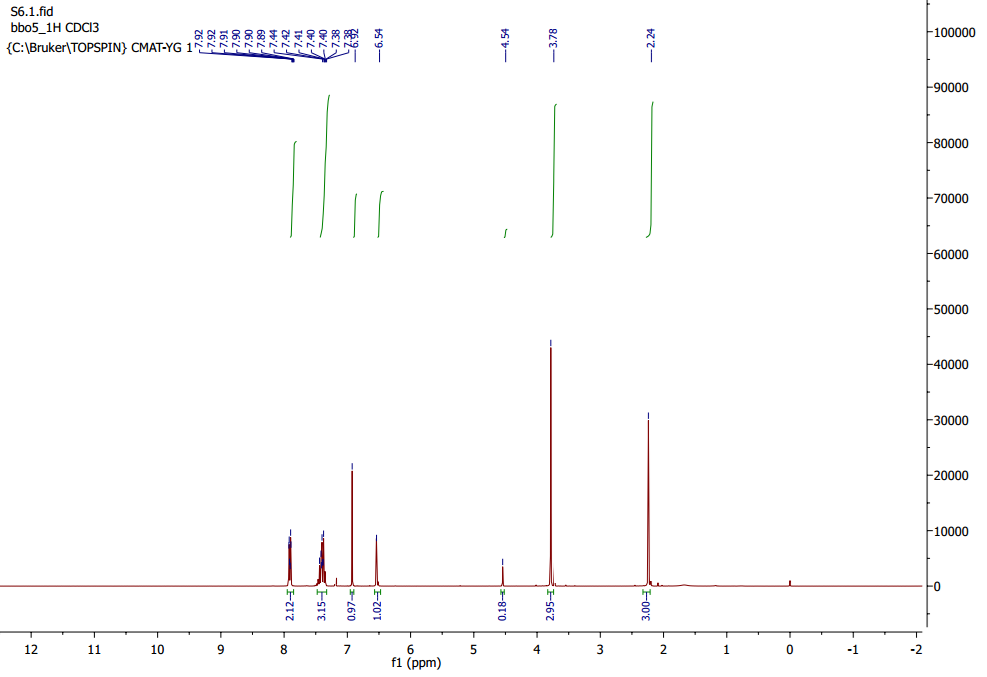
**

**Figure S1.** ^1^H NMR spectrum of **1**

**Figure S2.** ^13^C NMR spectrum of **1**

**Figure S3.** ^13^C NMR-DEPTQ-135 spectrum of **1**

**
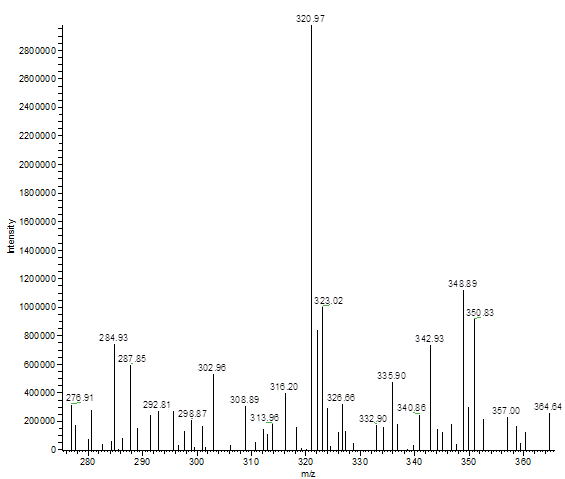
**

**Figure S4.** Mass spectrum of **1**


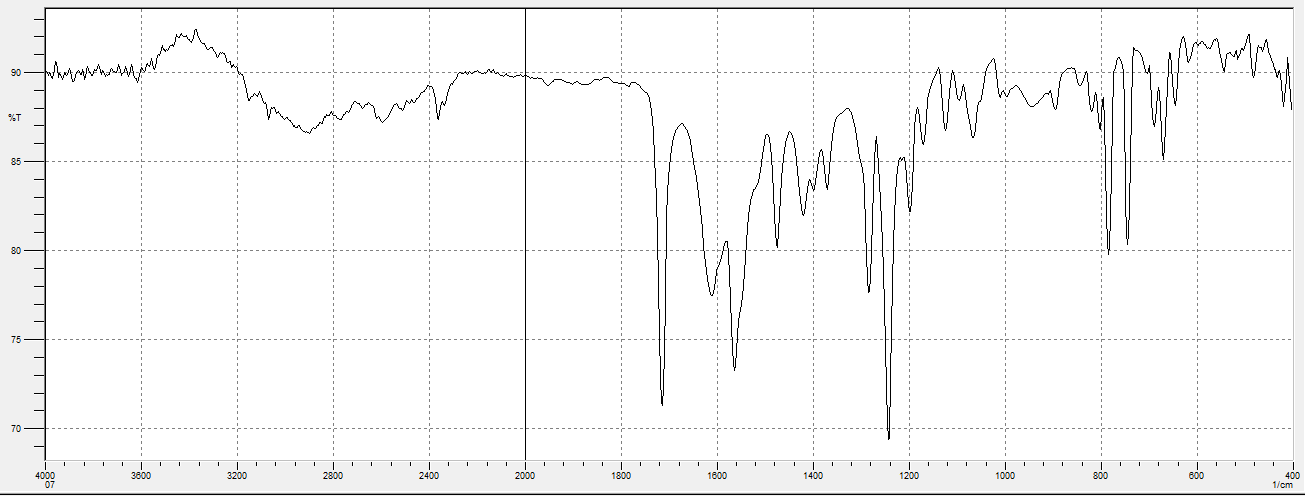


**Figure S5.** FT-IR spectrum of **1**

**Figure S6.** UV-Vis spectrum of **1**
